# Supplementary material for: Development and validation of the MY-VEG-FFQ: A modular web-based food-frequency questionnaire for vegetarians and vegans
Source: PLoS One. 2024 Apr 16;19(4):e0299515. doi: 10.1371/journal.pone.0299515 (PMC11020715; doi:10.1371/journal.pone.0299515)
Supplement: S1 Fig — (PDF) [file pone.0299515.s001.pdf]

Figure S1: Skipping algorithm in the My-VEG-FFQ*

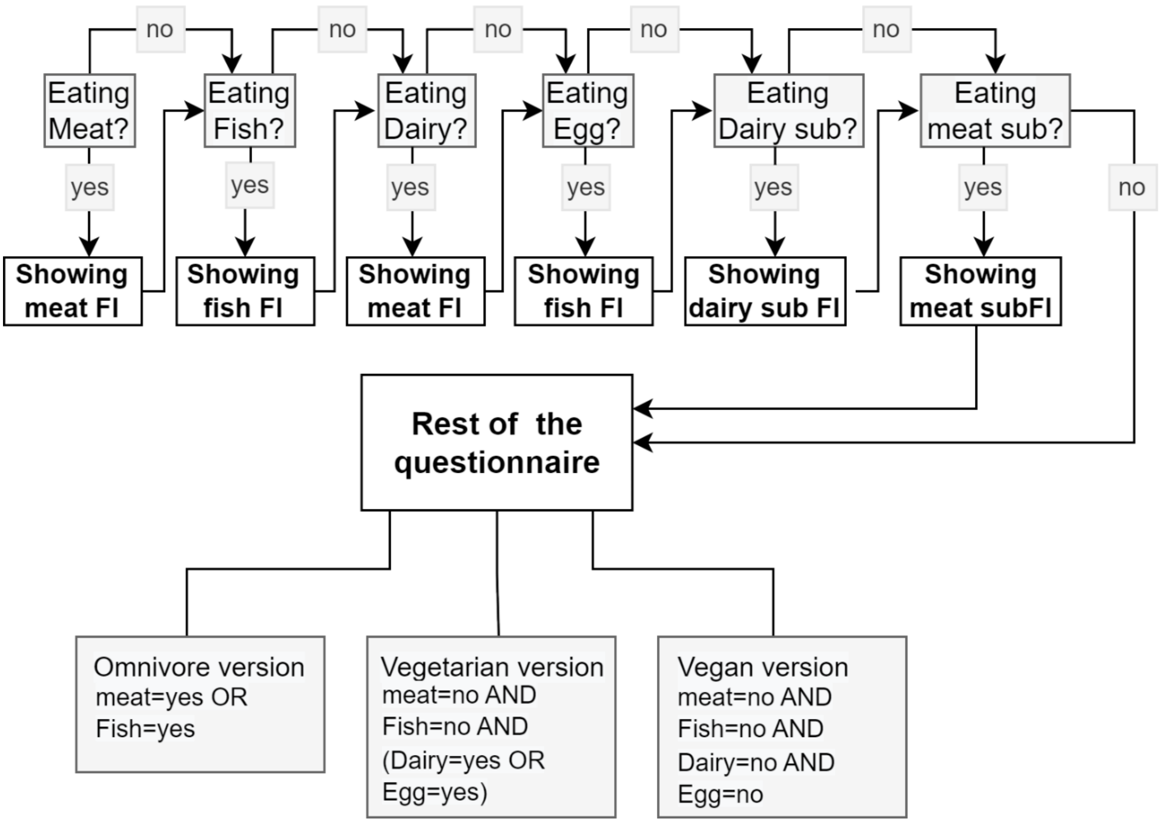

FFQ= Food-Frequency Questionnaire; F= Food items; Sub=substitutes

The skipping algorithm is based on our asking questions at the onset of the survey about the respondents' habitual consumption of meat, fish, eggs, dairy products, meat substitutes, and dairy substitutes. Besides displaying or hiding specific food groups – as per their answers, the questionnaire had three different versions, based on these questions. The vegetarian and vegan questionnaires had more options than the omnivore version for certain legume dishes and for spreads made from nuts and seeds. The vegan version also included vegan options for foods that typically contain dairy or eggs, such as baked goods and cakes.
