# Supplementary material for: Development and validation of the MY-VEG-FFQ: A modular web-based food-frequency questionnaire for vegetarians and vegans
Source: PLoS One. 2024 Apr 16;19(4):e0299515. doi: 10.1371/journal.pone.0299515 (PMC11020715; doi:10.1371/journal.pone.0299515)
Supplement: S2 Fig — (PDF) [file pone.0299515.s002.pdf]

Figure S2. Study flowchart.

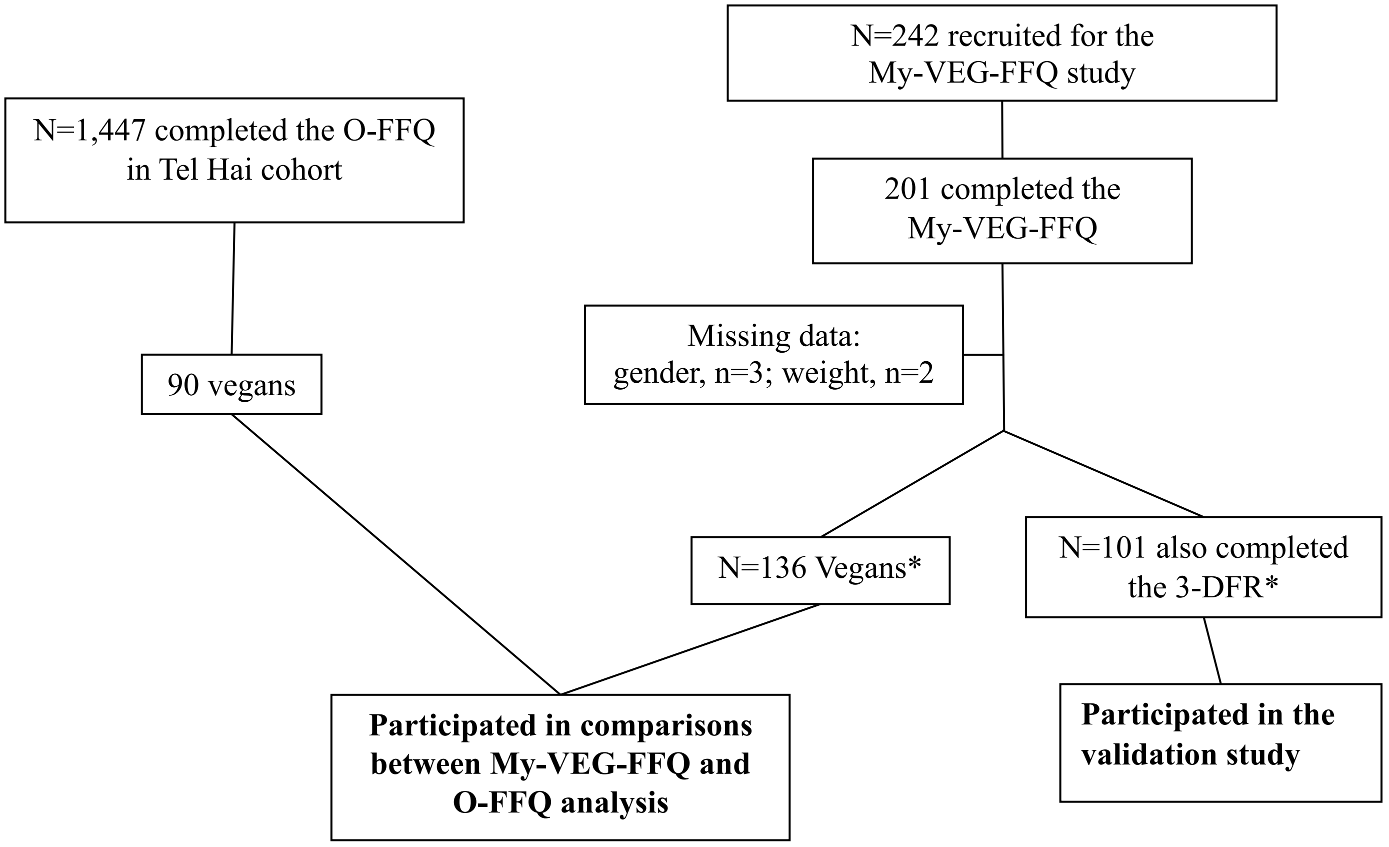

* Of the 136 vegans who completed the My-VEG-FFQ, 77 also completed the 3-DFR, thereby participating in both analyses; 3-DFR= three-day food record; FFQ= Food-Frequency Questionnaire.
