# Supplementary material for: Development and validation of the MY-VEG-FFQ: A modular web-based food-frequency questionnaire for vegetarians and vegans
Source: PLoS One. 2024 Apr 16;19(4):e0299515. doi: 10.1371/journal.pone.0299515 (PMC11020715; doi:10.1371/journal.pone.0299515)
Supplement: S3 Fig — (PDF) [file pone.0299515.s003.pdf]

Figure S3. Bland-Altman plots of individual differences in nutrient intake between the My-Veg-FFQ and the three-day food record (based on averages).

Macro-nutrients

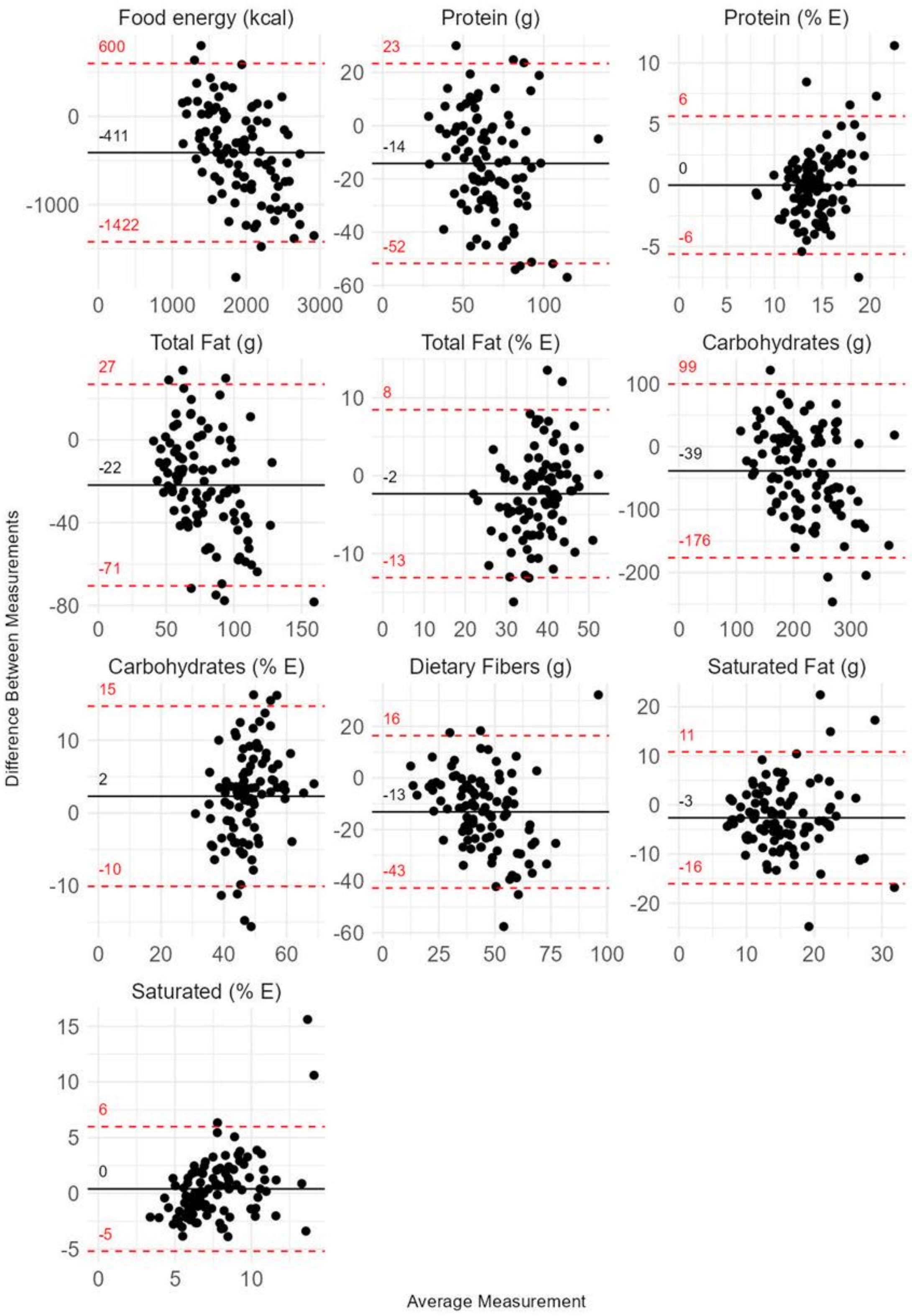

Micro-nutrients

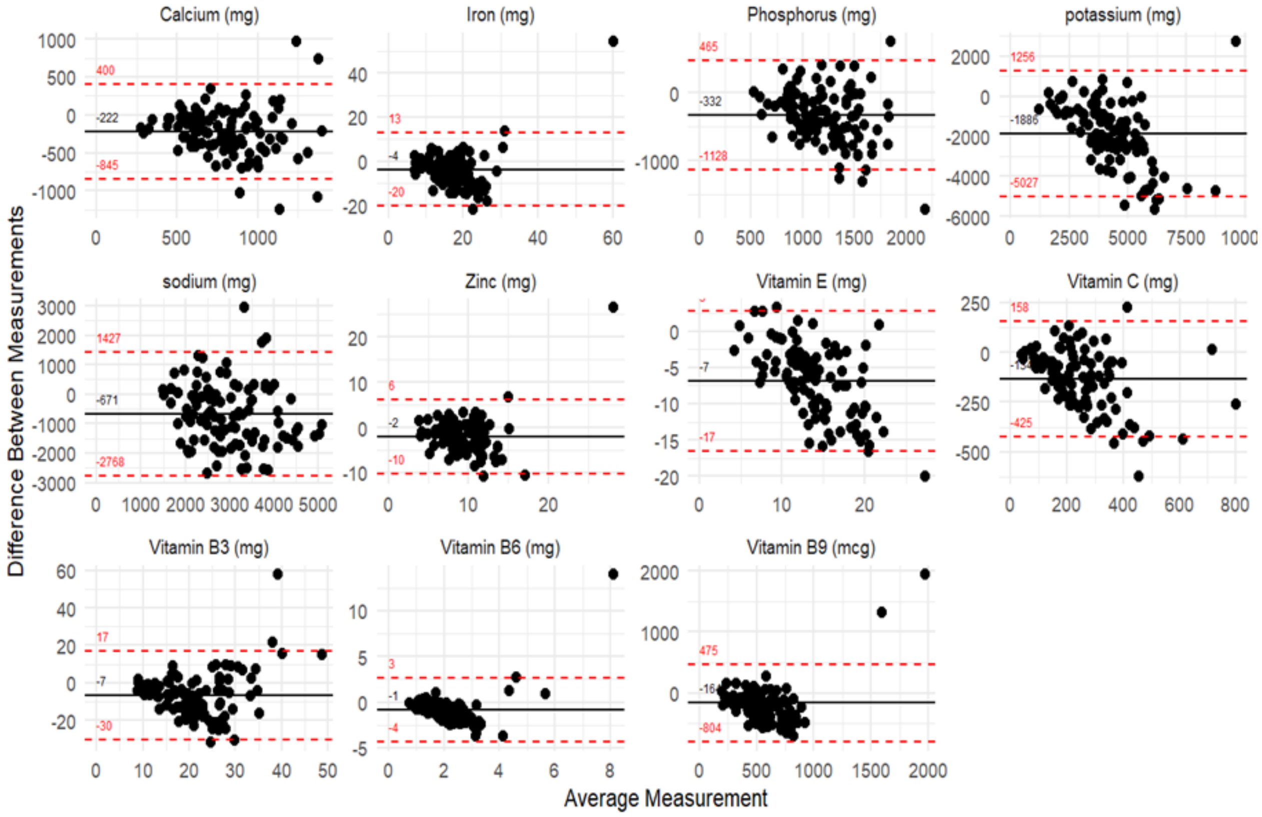

The dotted red lines represent the upper and lower 95% limits of agreement, while the solid black lines represent the mean agreement.
