# Supplementary material for: Development and validation of the MY-VEG-FFQ: A modular web-based food-frequency questionnaire for vegetarians and vegans
Source: PLoS One. 2024 Apr 16;19(4):e0299515. doi: 10.1371/journal.pone.0299515 (PMC11020715; doi:10.1371/journal.pone.0299515)
Supplement: S4 Fig — (PDF) [file pone.0299515.s004.pdf]

Figure S4. Comparison of correlations (R^2) for different dietary components between the current study and validation studies of FFQ questionnaires from previous studies.

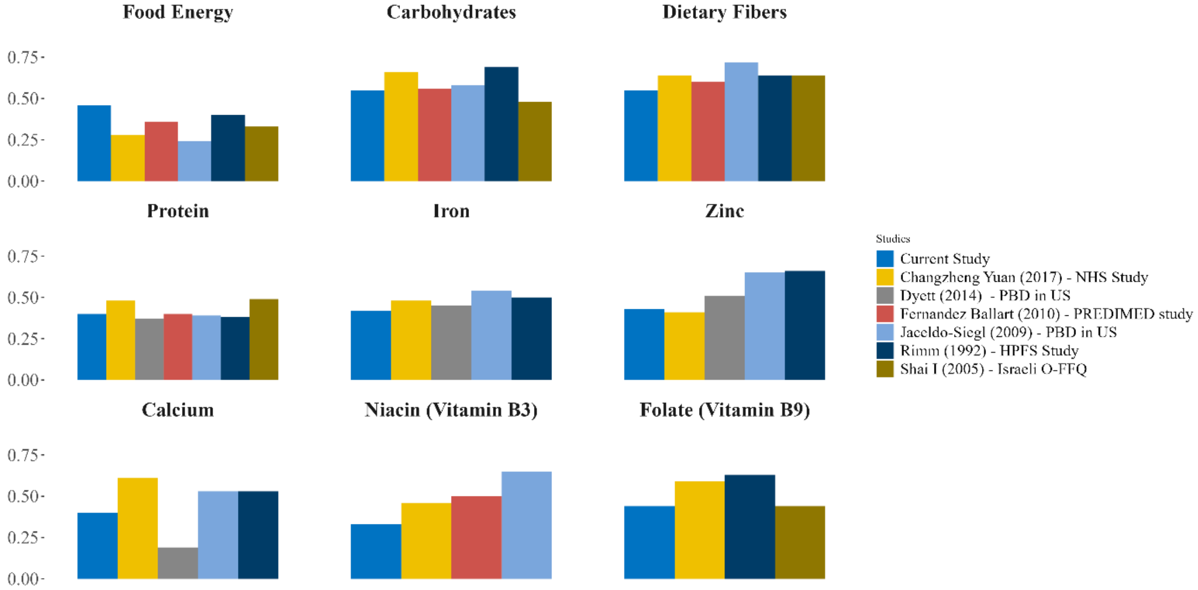

FFQ: Food-Frequency Questionnaire; NHS: FFQ used in the Nurses' Health Study; PBD: FFQ for population who consume Plant based diet; PREDIMED: FFQ used in the Prevención con Dieta Mediterránea study; HPFS: FFQ used in the Health Professionals Follow-up Study; O-FFQ= Original FFQ used in Israel.

Studies included in the figure:

Yuan C, Spiegelman D, Rimm EB, Rosner BA, Stampfer MJ, Barnett JB, et al. Validity of a Dietary Questionnaire Assessed by Comparison With Multiple Weighed Dietary Records or 24-Hour Recalls. American Journal of Epidemiology. 2017;185: 570–584. doi:10.1093/aje/kww104

Dyett P, Rajaram S, Haddad EH, Sabate J. Evaluation of a validated food frequency questionnaire for self-defined vegans in the United States. Nutrients. 2014;6: 2523–2539. doi:10.3390/nu6072523

Fernández-Ballart JD, Piñol JL, Zazpe I, Corella D, Carrasco P, Toledo E, et al. Relative validity of a semi-quantitative food-frequency questionnaire in an elderly Mediterranean population of Spain. Br J Nutr. 2010;103: 1808–1816. doi:10.1017/S0007114509993837

Jaceldo-siegl K, Knutsen SF, Sabate J, Chan J, Herring RP, Butler TL, et al. Validation of nutrient intake using an FFQ and repeated 24 h recalls in black and white subjects of the Adventist Health. Public Health Nutrition. 2009;13: 812–819. doi:10.1017/S1368980009992072

Shai I, Rosner BA, Shahar DR, Vardi H, Azrad AB, Kanfi A, et al. Dietary evaluation and attenuation of relative risk: multiple comparisons between blood and urinary biomarkers, food frequency, and 24-hour recall questionnaires: the DEARR study. The Journal of nutrition. 2005;135: 573–9. doi:10.1093/jn/135.3.573

Rimm EB, Giovannucci EL, Stampfer MJ, Colditz GA, Litin LB, Willett WC. Reproducibility and validity of an expanded self-administered semiquantitative food frequency questionnaire among male health professionals. American Journal of Epidemiology. 1992;135: 1114–1126. doi:10.1093/oxfordjournals.aje.a116211
