# Supplementary material for: Development and validation of the MY-VEG-FFQ: A modular web-based food-frequency questionnaire for vegetarians and vegans
Source: PLoS One. 2024 Apr 16;19(4):e0299515. doi: 10.1371/journal.pone.0299515 (PMC11020715; doi:10.1371/journal.pone.0299515)
Supplement: S1 Table — (PDF) [file pone.0299515.s005.pdf]

Table S1. Daily consumption of nutrients as estimated by the 3-DFR and MY-VEG-FFQ – crude comparison (full).

Nutrient	3-DFR (N=101) Mean (SD)	MY-VEG-FFQ (N=101) Mean (SD)	Diff.	95% CI	p-value [a]
Food energy (kcal)	1,722 (421)	1,912 (518)	-190	-321, -59	0.005
Carbohydrates (g)	201.1 (56.4)	213.4 (63.6)	-12.3	-29.0, 4.4	0.15
Carbohydrates (% E)	49.2 (8.3)	46.7 (6.7)	2.5	0.4, 4.6	0.021
Protein (g)	59.4 (19.4)	68.6 (21.3)	-9.2	-14.9, -3.6	0.001
Protein (% E)	14.5 (3.2)	15.0 (2.4)	-0.6	-1.4, 0.2	0.15
Total Fat (g)	66.6 (22.4)	78.1 (26.5)	-11.5	-18.4, -4.7	<0.001
Total Fat (% E)	36.4 (7.3)	38.3 (5.8)	-1.9	-3.7, 0.0	0.044
Saturated Fat (g)	14.5 (7.2)	15.1 (6.0)	-0.6	-2.5, 1.2	0.5
Saturated (% E)	8.0 (4.0)	7.4 (2.2)	0.6	-0.3, 1.5	0.2
Dietary Fibers (g)	39 (15)	46 (16)	-7.8	-12, -3.5	<0.001
Cholesterol (mg)	33.9 (80.2)	27.1 (54.7)	6.8	-12.2, 25.9	0.5
Calcium (mg)	676.1 (273.3)	839.1 (287.7)	-163.0	-240.8, -85.1	<0.001
Iron (mg)	15.5 (9.1)	17.1 (5.6)	-1.6	-3.7, 0.5	0.14
Phosphorus (mg)	1,046.2 (344.4)	1,238.0 (386.1)	-191.9	-293.4, -90.3	<0.001
potassium (mg)	3,280.5 (1,161.6)	4,344.4 (1,514.6)	-1,063.9	-1,438.6, -689.2	<0.001
sodium (mg)	2,632.2 (964.9)	2,694.4 (835.8)	-62.1	-312.6, 188.4	0.6
Zinc (mg)	8.5 (4.3)	9.4 (3.0)	-0.9	-1.9, 0.1	0.084
Vitamin E (mg)	10.3 (3.9)	14.8 (5.1)	-4.5	-5.7, -3.2	<0.001
Vitamin C (mg)	182.0 (136.3)	263.2 (150.9)	-81.2	-121.1, -41.3	<0.001

Nutrient	3-DFR (N=101) Mean (SD)	MY-VEG-FFQ (N=101) Mean (SD)	Diff.	95% CI	p-value [a]
Vitamin B3 (mg)	18.2 (11.4)	22.2 (7.8)	-4.0	-6.7, -1.3	0.004
Vitamin B6 (mg)	1.9 (1.9)	2.3 (0.8)	-0.4	-0.8, 0.0	0.079
Vitamin B9 (mcg)	466.5 (278.8)	573.6 (201.6)	-107.1	-174.7, -39.6	0.002

3-DFR= three-day food record; CI = confidence interval; Diff= Difference; FFQ= Food-Frequency Questionnaire.

[a] Welch two sample t-test.

s
