# Supplementary material for: Development and validation of the MY-VEG-FFQ: A modular web-based food-frequency questionnaire for vegetarians and vegans
Source: PLoS One. 2024 Apr 16;19(4):e0299515. doi: 10.1371/journal.pone.0299515 (PMC11020715; doi:10.1371/journal.pone.0299515)
Supplement: S2 Table — (PDF) [file pone.0299515.s006.pdf]

Table S2. Correlations between the MY-VEG-FFQ and the three-day food record.

Nutrients	R^2 (Pearson correlation crude)	R^2 (Pearson correlation adjusted) [a]
Carbohydrates (g)	0.35	0.55
Carbohydrates (% E)	0.56	0.56
Food energy (kcal)	0.46	0.46
Protein (g)	0.47	0.40
Protein (% E)	0.41	0.41
Saturated fat (g)	0.39	0.41
Saturated (% E)	0.40	0.40
Dietary fibers (g)	0.51	0.55
Total fat (g)	0.51	0.53
Total fat (% E)	0.54	0.54
Cholesterol (mg)	0.64	0.63
Calcium (mg)	0.47	0.40
Iron (mg)	0.44	0.42
Phosphorus (mg)	0.40	0.33
Potassium (mg)	0.52	0.48
Sodium (mg)	0.39	0.36
Zinc (mg)	0.42	0.43
Vitamin E (mg)	0.45	0.25
Vitamin C (mg)	0.40	0.38
Vitamin B3 (mg)	0.33	0.33
Vitamin B6 (mg)	0.38	0.37
Vitamin B9 (mcg)	0.47	0.44

FFQ= Food-Frequency Questionnaire,
[a] Quantities were log-transformed and assessed per 1000 Kcal, except for energy and % macro-nutrients from energy, where quantities where only log-transformed.
