# Supplementary material for: Development and validation of the MY-VEG-FFQ: A modular web-based food-frequency questionnaire for vegetarians and vegans
Source: PLoS One. 2024 Apr 16;19(4):e0299515. doi: 10.1371/journal.pone.0299515 (PMC11020715; doi:10.1371/journal.pone.0299515)
Supplement: S3 Table — (PDF) [file pone.0299515.s007.pdf]

Table S3. Cross-classification of quartiles of macro-nutrients intake derived from My-VEG-FFQ vs. three-day food record.

Nutrients	Fully Agree	±Q	Fully±Q	±2Q	±3Q	Kw*
Food energy (kcal)	32.7	46.5	79.2	15.8	5.0	0.39
Protein (g)	35.6	43.6	79.2	18.8	2.0	0.46
Protein (% E)	31.7	44.6	76.3	18.8	5.0	0.35
Total fat (g)	36.6	43.6	80.2	17.8	2.0	0.47
Total fat (% E)	47.5	30.7	78.2	16.8	5.0	0.43
Carbohydrates (g)	31.7	41.6	73.3	20.8	5.9	0.29
Carbohydrates (% E)	45.5	36.6	82.1	12.9	5.0	0.47
Dietary fibers (g)	39.6	39.6	79.2	18.8	2.0	0.47
Saturated fat (g)	36.6	43.6	80.2	15.8	4.0	0.43
Saturated (% E)	36.6	43.6	80.2	13.9	5.9	0.39
Cholesterol (mg)	37.6	37.6	75.2	22.8	2.0	0.42
Calcium (mg)	39.6	40.6	80.2	14.9	5.0	0.43
Iron (mg)	32.7	40.6	73.3	23.8	3.0	0.36
Phosphorus (mg)	33.7	39.6	73.3	24.8	2.0	0.38
Potassium (mg)	43.6	35.6	79.2	14.9	5.9	0.41
Sodium (mg)	36.6	38.6	75.2	17.8	6.9	0.32
Zinc (mg)	29.7	45.5	75.2	20.8	4.0	0.35
Vitamin E (mg)	36.6	40.6	77.2	19.8	3.0	0.42
Vitamin C (mg)	36.6	44.6	81.2	11.9	6.9	0.39
Vitamin B3 (mg)	29.7	49.5	79.2	12.9	7.9	0.32
Vitamin B6 (mg)	41.6	32.7	74.3	20.8	5.0	0.36
Vitamin B9 (mcg)	41.6	35.6	77.2	20.8	2.0	0.46

FFQ= Food-Frequency Questionnaire; Q = quartiles; fully agree = % of cases cross-classified into the same quartile; ±Q1/2/3: % of cases cross-classified 1/2/3 quartiles apart, respectively.
* Weighted kappa statistics for cross-classified of the total population.
