# Supplementary material for: Development and validation of the MY-VEG-FFQ: A modular web-based food-frequency questionnaire for vegetarians and vegans
Source: PLoS One. 2024 Apr 16;19(4):e0299515. doi: 10.1371/journal.pone.0299515 (PMC11020715; doi:10.1371/journal.pone.0299515)
Supplement: S4 Table — (PDF) [file pone.0299515.s008.pdf]

Table S4. Daily consumption of nutrients of vegans, estimated by the O-FFQ and the MY-VEG-FFQ.

Nutrient	MY-VEG-FFQ (N=136) Mean (SD)	O-FFQ (N=90) Mean (SD)	P value [a]	Adjusted p value [b]
Food energy (kcal)	1,963 (657)	1,698 (823)	<0.001	<0.001
Protein (g)	71.1 (27.1)	51.2 (24.3)	<0.001	<0.001
Protein (% E)	15.2 (2.4)	13.0 (2.9)	<0.001	<0.001
Total Fat (g)	79.6 (30.2)	57.6 (28.5)	<0.001	<0.001
Total Fat (% E)	38.0 (5.5)	32.8 (7.9)	<0.001	<0.001
Carbohydrates (g)	218.3 (77.5)	219.1 (122.2)	0.3	0.15
Carbohydrates (% E)	46.8 (6.7)	54.2 (9.5)	<0.001	<0.001
Dietary Fibers (g)	48.5 (17.8)	42.3 (21.2)	0.001	0.001
Saturated Fat (g)	14.2 (6.0)	10.3 (5.3)	<0.001	<0.001
Saturated Fat (% E)	6.8 (1.5)	6.0 (1.9)	<0.001	<0.001
Cholesterol (mg)	2.1 (8.5)	35.2 (67.7)	<0.001	<0.001
Calcium (mg)	823.7 (329.9)	720.3 (336.5)	0.005	0.011
Iron (mg)	18.2 (6.5)	14.0 (7.2)	<0.001	<0.001
Phosphorus (mg)	1,220.6 (434.6)	1,079.7 (506.7)	0.002	0.001

Potassium (mg)	4,420.7 (1,681.2)	4,206.5 (2,023.0)	0.2	0.2
Sodium (mg)	2,830.3 (1,098.7)	2,538.6 (1,605.3)	0.003	<0.001
Zinc (mg)	9.5 (3.4)	8.0 (3.7)	<0.001	<0.001
Vitamin E (mg)	14.4 (5.2)	14.7 (7.3)	0.8	>0.9
Vitamin C (mg)	258.7 (135.6)	292.7 (195.8)	0.4	0.3
Vitamin B3 (mg)	22.2 (9.3)	23.3 (11.4)	0.7	0.7
Vitamin B6 (mg)	2.3 (0.9)	2.6 (1.4)	0.2	0.2
Vitamin B9 (mcg)	585.3 (219.8)	515.9 (255.8)	0.006	0.006

FFQ = Food-Frequency Questionnaire; [a] ANCOVA (log transformed) | [b] ANCOVA (log transformed) adjusted for sex and age.
