# Supplementary material for: Development and validation of the MY-VEG-FFQ: A modular web-based food-frequency questionnaire for vegetarians and vegans
Source: PLoS One. 2024 Apr 16;19(4):e0299515. doi: 10.1371/journal.pone.0299515 (PMC11020715; doi:10.1371/journal.pone.0299515)
Supplement: S5 Table — (PDF) [file pone.0299515.s009.pdf]

Table S5. Daily consumption of various food items estimated by the O-FFQ for omnivores and by the My-VEG-FFQ for vegetarians and vegans.

Food item (gr/day)	Answered the MY-VEG-FFQ (N=136) Mean (SD)	Answered the O-FFQ (N=90) Mean (SD)	p-value [a]	p-value [b]
Vegetables	536.3 (308.7)	632.8 (384.0)	0.05	0.013
Fruits	325.7 (332.3)	436.3 (632.8)	0.10	0.042
Whole grains, including whole-wheat bread & brown rice	107.4 (81.7)	148.2 (125.9)	0.083	0.3
Refined grains, including white bread & rice	62.7 (56.6)	59.3 (62.7)	0.9	0.3
Pastries including pizza	18.7 (27.5)	6.9 (7.9)	0.001	<0.001
Potatoes: baked, boiled, or fried	30.9 (41.7)	30.3 (50.9)	0.5	0.2
Legumes & mixed dishes without meat	244.3 (146.5)	192.8 (198.7)	<0.001	<0.001
Meat analog, including tofu & vegetable patties	167.9 (140.1)	41.9 (50.1)	<0.001	<0.001
Meat analog, including vegetable patties	18.6 (23.0)	-		-
Tofu soy protein & seitan dishes	149.2 (136.2)	-		-
Milk analog including soy beverages	265.7 (270.7)	114.7 (152.6)	<0.001	<0.001
Nuts & seeds	46.5 (31.6)	25.4 (27.4)	<0.001	<0.001
Olive oil & table olives	8.4 (9.5)	9.0 (10.0)	>0.9	0.7
Other oils	1.8 (2.0)	1.3 (2.4)	0.006	0.009
Avocado	20.6 (25.7)	11.2 (17.6)	0.002	0.015
Snacks & sweets	31.3 (31.0)	27.6 (35.5)	0.074	0.2
Fruit juice	18.3 (55.3)	17.9 (31.2)	0.63	0.9
Soft drinks	52.1 (136.8)	82.2 (265.0)	0.4	0.6

**Food item (gr/day)**	**Answered the MY-VEG-FFQ (N=136) Mean (SD)**	**Answered the O-FFQ (N=90) Mean (SD)**	**p-value [a]**	**p-value [b]**
Tea & coffee	470.4 (382.2)	514.9 (433.9)	0.5	0.8
Alcoholic beverages	41.7 (67.1)	73.6 (266.6)	0.3	0.7
Water	1,081.8 (390.9)	1,228.2 (371.6)	0.8	0.66
Animal-based food items	0.1 (1.3)	8.4 (56.0)	0.009	0.009

FFQ= Food-Frequency Questionnaire; O-FFQ = Original FFQ

[a] ANCOVA (log transformed) | [b]ANCOVA (log transformed) adjusted for sex and age
