# Supplementary material for: Development and validation of the MY-VEG-FFQ: A modular web-based food-frequency questionnaire for vegetarians and vegans
Source: PLoS One. 2024 Apr 16;19(4):e0299515. doi: 10.1371/journal.pone.0299515 (PMC11020715; doi:10.1371/journal.pone.0299515)
Supplement: S1 File — (PDF) [file pone.0299515.s010.pdf]

List of food items in the MY-VEG FFQ*

Food Item	Serving	Displayed for?**
**Milk 0-2% fat**	1 cup	Milk & dairy eaters
**Milk >2% fat**	1 cup	Milk & dairy eaters
**Chocolate & other flavored milk**	1 cup	Milk & dairy eaters
**Farmer or Cottage cheese 0-5% fat**	1 heaping spoonful	Milk & dairy eaters
**Farmer or Cottage cheese >5% fat**	1 heaping spoonful	Milk & dairy eaters
**Skimmed yogurt 0.5-2% fat without sugar**	1 container	Milk & dairy eaters
**Skimmed yogurt >2% fat without sugar**	1 container	Milk & dairy eaters
**Skimmed yogurt 0.5-2% fat with added sugar and flavors**	1 container	Milk & dairy eaters
**Skimmed yogurt >2% fat with added sugar and flavors**	1 container	Milk & dairy eaters
**Low fat hard cheese <9% fat**	1 slice	Milk & dairy eaters
**Hard cheese >9% fat**	1 slice	Milk & dairy eaters
**Brined cheese 0-5% fat**	1 slice/10 cubes	Milk & dairy eaters
**Brined cheese >5% fat**	1 slice/10 cubes	Milk & dairy eaters
**Labane - Strained yogurt**	1 tablespoon	Milk & dairy eaters
**Soy beverage low sugar**	1 cup	Milk & dairy substitutes eaters
**Soy beverage with added sugar / flavored**	1 cup	Milk & dairy substitutes eaters
**Other plant-based drink (rice, oats, almonds etc)**	1 cup	Milk & dairy substitutes eaters
**Plant-based yogurt/desserts with added sugar or fruits**	1 container	Milk & dairy substitutes eaters
**Plant-based yogurt low sugar/ diet**	1 container	Milk & dairy substitutes eaters
**Soy and coconut hard cheese**	1 slice	Milk & dairy substitutes eaters
**Nuts cheese (hand-made/bought)**	1 slice / 1 heaping spoonful	Milk & dairy substitutes eaters
**Soy cheese spread**	1 heaping spoonful	Milk & dairy substitutes eaters
**Plant-based cream (from soy, rice, or oats)**	½ cup (100 ml)	Milk & dairy substitutes eaters
**Nutritional yeast**	1 tablespoon	Vegans
**Omelets and scrambled egg**	1 egg	Eggs eaters
**Hard-boiled egg**	1 egg	Eggs eaters
**Chicken/turkey-fried steak**	1 unit (100-120 grams)	Meat (beef and poultry) eaters
**Chicken nuggets**	1 medium unit or 4-5 small units	Meat (beef and poultry) eaters

**Poultry, turkey, goose, duck**	1 medium serving / 1 drumstick	Meat (beef and poultry) eaters
**Red meat (beef and lamb)**	1 medium serving	Meat (beef and poultry) eaters
**Shawarma in pitta bread**	1 serving	Meat (beef and poultry) eaters
**Meat cutlets**	2 small cutlets or one medium	Meat (beef and poultry) eaters
**Mixed dishes with meat (stuffed vegetables, stews etc)**	1 serving/1 plate	Meat (beef and poultry) eaters
**Organ meats**	1 serving/1 plate	Meat (beef and poultry) eaters
**Pastrami (turkey/poultry)**	1 slice	Meat (beef and poultry) eaters
**Processed meat (sausages, hotdog etc)**	1 hot dog / 2 slices	Meat (beef and poultry) eaters
**Chicken soup and meat broth**	1 bowl	Meat (beef and poultry) eaters
**Fish (fried or baked)**	1 medium serving/ 2 cutlets	Fish eaters
**Tuna (including tuna salad)**	1 heaping spoonful	Fish eaters
**Pickled fish**	1 slice / 1 teaspoon	Fish eaters
**Legumes cutlet**	1 medium cutlet or 2 small cutlets	Meat substitutes eaters
**Soy chunk and soy bolognese**	3 heaping spoonsful	Meat substitutes eaters
**Mat alternatives**	1 plant-based hamburger/fried steak, or 2 hotdogs	Meat substitutes eaters***
**Vegetables cutlets**	1 medium cutlet or 2 small cutlets	Meat substitutes eaters***
**Seitan dishes**	1 plate	Meat substitutes eaters
**White bread/ pitta bread /bread roll**	1 slice / half pitta bread	Everyone
**Whole wheat bread/ pitta bread /bread roll**	1 slice / half pitta bread	Everyone
**Diet wheat bread/ pitta bread /bread roll**	1 slice / half pitta bread	Everyone
**Pizza**	1 slice	Omnivore and vegetarian
**Vegan pizza**	1 slice	Vegans
**Quiche or pie with vegetables and dairy**	1 slice / medium unit	Omnivore and vegetarian
**Vegan Quiche or pie with vegetables**	1 slice / medium unit	Vegans

**Puff pastry (burek, Malawach etc)**	1 medium unit or half large one	Everyone***
**Pasta from white wheat**	1 cup (cooked)	Everyone
**Pasta from whole wheat**	1 cup (cooked)	Everyone
**White rice**	1 cup (cooked)	Everyone
**Whole rice**	1 cup (cooked)	Everyone
**Other grains (burgul, buckwheat, corn)**	1 cup (cooked)	Everyone
**Quinoa (including quinoa salad)**	1 cup (cooked)	Vegetarian & vegans
**Baked or boiled potatoes including puree**	1 serving	Everyone***
**French fries**	1 serving	Everyone
**Ready-to-eat breakfast cereals, such as cornflakes, granola and cereal bars**	1 bowl or one snack	Everyone***
**Oatmeal and other porridges**	1 bowl	Everyone***
**Cooked legumes eaten separately or part of a dish**	½ cup (cooked)	Omnivore
**Falafel with pitta bread**	1 serving	Everyone
**Hummus and tahini**	1heaping spoonful	Omnivore
**Hummus and tahini**	1 plate	Vegetarians & vegans
**Legume soup**	1 bowl	Everyone
**Vegetarian dishes including stuffed vegetables**	1 plate / 2 units of stuffed vegetables	Omnivore
**Legumes eaten separately**	1 cup (cooked)	Vegetarians & vegans
**Dishes based on tofu**	1 plate with approximately 150 grams of tofu)	Vegetarians & vegans
**Legume spreads**	1 heaping spoonful	Vegetarians & vegans
**Legume-based pasta**	1 cup (cooked)	Vegetarians & vegans
**Vegan omelet (based on chickpeas/lentils flour)**	1 omelet made with ½ cup legume flour	Vegans
**Vegetable salads**	1 serving / 1 cup	Everyone
**Tomato (fresh or cooked)**	1 large unit or 5-10 small ones	Everyone
**Tomato sauce or ketchup**	1 spoonful	Everyone
**Cucumbers**	1 unit	Everyone
**Bell pepper**	1 unit	Everyone
**Zucchini and eggplant (baked and fried)**	1 serving / plate	Everyone
**Broccoli, cauliflower, brussels sprout and vegetable mix**	½ cup	Everyone
**Cabbage salads**	½ cup / 1 small bowl	Everyone***
**Lettuce and mesclun**	1 cup	Everyone

**Carrot (fresh or cooked)**	1 unit	Everyone
**Green peas, green and yellow beans**	1 cup	Everyone
**Sweet potatoes, pumpkins**	1 serving	Everyone
**Green leaves**	1 cup	Vegetarians & vegans
**Other vegetables including mushrooms**	1 serving / 1 cup	Everyone
**Avocado including avocado salad**	½ unit / 2 heaping spoonful	Everyone
**Cooked vegetable salad**	1 heaping spoonful	Everyone***
**Pickled vegetables**	1 unit	Everyone
**Olives**	10 units	Everyone
**Lemon juice**	Juice from ½ lemon	Everyone
**Vegetable soup**	1 bowl	Everyone
**Legume dishes**	1 plate	Vegetarians & vegans
**Stir-fry dishes with tofu or seitan**	1 plate	Vegetarians & vegans
**Legume soup**	1 bowl	Everyone
**Grains soup**	1 bowl	Everyone
**Seaweed dishes (sushi, wakame salad)**	1 sushi roll/ 1 plate of wakame salad	Vegans
**Apple and pear**	1 unit	Everyone
**Orange and other citrus fruits**	1 orange / 3 clementine	Everyone
**Banana**	1 unit	Everyone
**Peach and plum**	1 peach or 2 plums	Everyone
**Grapes and raisins**	½ cup of grapes	Everyone
**Melon**	1 medium slice	Everyone
**Watermelon**	1 large slice / 2 small slices	Everyone
**Other fruits and fruit salad**	½ large units or 1 cup	Everyone
**Fruit juice (orange etc.)**	1 cup	Everyone
**Dried fruit**	4 medium units	Everyone
**Nuts, peanuts and almond**	25 grams / ½ cup	Everyone
**Seeds**	25 grams / ½ cup	Everyone
**Flax seeds**	1 teaspoon	Vegetarians & vegans
**Nut butters, including peanut butter**	1 teaspoon	Vegetarians & vegans
**Whole tahini**	1 teaspoon	Vegetarians & vegans
**White tahini**	1 teaspoon	Vegetarians & vegans
**Olive oil**	1 teaspoon	Everyone
**Other vegetable oils (canola, soy, corn etc)**	1 teaspoon	Everyone
**Vegan butter and margarine**	1 teaspoon	Everyone

**Butter**	1 teaspoon	Omnivores and vegetarians
**Mayonnaise and mayonnaise sauces**	1 teaspoon	Omnivore and vegetarians
**Vegan mayonnaise and mayonnaise sauces**	1 teaspoon	Vegans
**Snacks**	1 small package (30 grams)	Everyone
**Crackers including rice crackers**	1 unit	Everyone
**Ice-cream (Dairy based)**	2 scoops / 1 Ice cream bar	Omnivore and vegetarians
**Non-dairy ice-cream**	2 scoops / 1 Ice cream bar	Everyone
**Diet ice-cream and frozen yogurt**	2 scoops / 1 Ice cream bar	Omnivore and vegetarians
**Vegan frozen yogurt**	2 scoops / 1 Ice cream bar	Vegans
**Popsicle or sorbet**	2 scoops / 1 ice pop	Everyone
**Vegan chocolate and chocolate snacks**	1 small bar / 4 chocolate cubes	Vegans
**Chocolate general**	1 small bar / 4 chocolate cubes	Omnivore and vegetarians
**Hard cookies**	1 large unit / 2 small units	Everyone***
**Waffles and chocolate chips cookies**	1 large unit / 2 small units	Everyone***
**Yeast cake**	1 slice	Everyone***
**Sponge cake (chocolate/fruits etc.)**	1 slice	Everyone***
**Cheese cake**	1 slice	Omnivore and vegetarians
**Vegan cheese cake**	1 slice	Vegans
**Arabic desserts (baklava, Halva)**	1 slice / 1 unit	Everyone***
**Honey, jam, maple syrup, chocolate spread**	1 teaspoon	Omnivore and vegetarians
**Jam, maple syrup, vegan chocolate spread**	1 teaspoon	Vegans
**Sugar**	1 teaspoon	Everyone
**Carbonated soft drinks**	1 cup	Everyone
**Diet soft drinks**	1 cup	Everyone
**Nectar fruit juice**	1 cup	Everyone
**Coffee**	1 cup	Everyone
**Tea**	1 cup	Everyone
**Beer**	1 can / ⅓ liter	Everyone
**Wine**	1 glass	Everyone
**Liquor, whiskey, vodka etc.**	30 ml / 1 shot glass	Everyone
**Water (tap or mineral)**	1 cup	Everyone

For each food item the participant needed to report on the frequency in which they consumed that specific item, as per the following scale:

Never or less than once per month
1–3 times per month
1–2 times per week
3–4 times per week
5–6 times per week
once per day
2–3 times per day
3–5 times per day
6 times per day or more

** See figure S1 for explanation of the skipping algorithm used for creating the semi-personalized FFQ

*** The items aggregated and calculated for this food item were slightly different for vegans compared to omnivores (for example a vegan version of pastry without dairy or cheese or salad with vegan mayonnaise instead of the regular one)
