# Supplementary material for: Development and validation of the MY-VEG-FFQ: A modular web-based food-frequency questionnaire for vegetarians and vegans
Source: PLoS One. 2024 Apr 16;19(4):e0299515. doi: 10.1371/journal.pone.0299515 (PMC11020715; doi:10.1371/journal.pone.0299515)
Supplement: Attachment — Submitted filename: Response to Reviewers.pdf [file pone.0299515.s013.pdf]

Ms. No. PONE-D-23-25091

Development and validation of the MY-VEG-FFQ: A modular web-based food-frequency questionnaire for vegetarians and vegans

To: **Diego A. Bonilla**
Academic Editor
PLOS ONE

Dear Dr. Bonilla,

We would like to thank the reviewers for their important contributions and the time taken to comment on our work. The reviewers' and the editor's comments and our respective responses are detailed below.

We hope that the manuscript in its current form is acceptable for publication in PLOS ONE journal.

Thank you for your reconsideration.

Sincerely,

Prof. Danit R Shahar, RD, PhD

The International center of health innovation & Nutrition

Department of Epidemiology, Biostatistics and Community Health Sciences

School of Public Health, Faculty of Health Sciences

Ben-Gurion University of the Negev, Israel

Point by point response:

Editor's comments:

If applicable, we recommend that you deposit your laboratory protocols in protocols.io to enhance the reproducibility of your results. Protocols.io assigns your protocol its own identifier (DOI) so that it can be cited independently in the future.

This research does not include any laboratory tests, therefore we have no protocol to deposit. We shall follow this recommendation in future projects if needed.

1. Please ensure that your manuscript meets PLOS ONE's style requirements, including those for file naming.

As requested, we modified our manuscript to meet PLOS ONE's style requirements. We also re-uploaded all the supplemental files to ensure that they follow the file naming instructions.

2. We suggest you thoroughly copyedit your manuscript for language usage, spelling, and grammar. If you do not know anyone who can help you do this, you may wish to consider employing a professional scientific editing service.

Upon resubmission, please provide the name of the colleague or the details of the professional service that edited your manuscript

The article has been professionally edited by Beverley Yohanan (www.business-letters.co.il), who has vast experience in academic articles. Please see enclosed letter from her.

3. PLOS requires an ORCID iD for the corresponding author in Editorial Manager on papers submitted after December 6th, 2016.
We added the corresponding author's ORCID ID

4. Please remove your figures from within your manuscript file, leaving only the individual TIFF/EPS image files, uploaded separately. These will be automatically included in the reviewers' PDF.

As requested, we removed the figures from the manuscript.

5. Please review your reference list to ensure that it is complete and correct. If you have cited papers that have been retracted, please include the rationale for doing so in the manuscript text, or remove these references and replace them with relevant current references. Any changes to the reference list should be mentioned in the rebuttal letter that accompanies your revised manuscript. If you need to cite a retracted article, indicate the article's retracted status in the References list and also include a citation and full reference for the retraction notice.

We reviewed the reference list and corrected references 19, 24, 26,27.

Reviewers' comments:

Reviewer's Responses to Questions

Comments to the Author

General:

3. Have the authors made all data underlying the findings in their manuscript fully available?

The PLOS Data policy requires authors to make all data underlying the findings described in their manuscript fully available without restriction, with rare exception (please refer to the Data Availability Statement in the manuscript PDF file). The data should be provided as part of the manuscript or its supporting information, or deposited to a public repository. For example, in addition to summary statistics, the data points behind means, medians and variance measures should be available. If there are restrictions on publicly sharing data—e.g. participant privacy or use of data from a third party—those must be specified.

Reviewer #1: Yes

Reviewer #2: No

The raw data underlying the findings described in the manuscript, is fully available as requested by the journal policy (file S2).

Reviewer #1:

In the statistical analysis section there is no mention of the use or not of tests that evaluate the type of distribution of the data. Since the authors use statistics to analyze data with Gaussian and non-Gaussian distribution, it would be appropriate to state the reason for this situation.

We appreciate the reviewer's comment. In response, we have included additional details regarding the distribution tests conducted to determine the appropriate analysis method. (Lines 167-170) Furthermore, we have added information about the transformations applied to the data on nutrients and food groups before conducting Pearson correlation tests. (lines 176)

Additionally, the comment has prompted us to consider applying a logarithmic transformation before using the ANCOVA test in the adjusted comparison with the Original FFQ. We believe that this adjustment enhances the statistical robustness of the test. We are grateful for highlighting this important point, and we thank you once again for your valuable feedback. (lines 179-180, Table 3, S4, S5)

Within the discussion and limitations of the study, no reference is made to the need or not to estimate the stability over time of the results. It is interesting to know if the authors consider that their instrument can provide stable and reproducible results over time or perhaps this is a limitation not addressed in the document.

We agree with the reviewer and addressed the issue regarding the lack of reproducible results over time. (lines 349-350).

While as stated in reference (36), a sample size around 100 subjects seems to be sufficient, I can't help but wonder why they didn't calculate their own sample size?

We appreciate the reviewer's valuable input. We have now incorporated the sample size calculation and relevant considerations into the study method section (lines 181-188). Additionally, we have included a comparison to the sample sizes of 130 FFQ validation studies reviewed by Cui Q et al in the limitation section (lines 343-344).

In Table 2, values appear in parentheses in the cells of the second and third column. What data are these? What contribution do these values make to the analysis of results?

We appreciate the reviewer's comment. The values within the parentheses represent the median values. After careful consideration, we have decided to remove these values from table 2. This decision was made because the median values closely aligned with the mean values and did not significantly contribute to the results. This change also eliminates unnecessary complexity. Consequently, we have removed the median values from Tables 3, S1, S4, and S5 as well.

Reviewer #2: The manuscript is notably intriguing as it introduces a fresh approach to nutritional control in vegan subjects, among other significant aspects.

However, it would be beneficial if the authors provided a more detailed explanation of the inclusion and exclusion criteria for participant subjects in the validation of the instrument. For example, were subjects with specific dietary restrictions excluded? Could subjects with any cognitive limitations (mild or otherwise) complete the questionnaire? Clarifying these aspects would help in better understanding the methodology used.

We value the reviewer's comment and have included the inclusion and exclusion criteria for participants in the method section (lines 90-91). It is important to note that we did not specify any limitations related to cognitive impairments. As indicated in Table 1, the majority of the participants had more than 12 years of schooling and the mean age was 39 years old suggesting that cognitive tests are not required for this group.

Furthermore, a thorough review of the tables is suggested, as at times they present figures with three decimal places, while in other instances, only two or even one decimal place is used. It would be advisable to standardize the format in accordance with the journal's style guidelines.

We have conducted a comprehensive review of the tables and have implemented the required modifications to maintain uniformity in the presentation of decimal places. Our approach aligns with that of Zheng et al, who published their research on the development and validation of a FFQ for children in PLOS One in 2020. In this approach, most means and percentage values are presented with one decimal place, except for energy values, which are displayed without decimal places. Correlations are depicted with two decimal places, following the article by Zheng et al [1] *(Tables 1-3, S1-S5).*

Regarding Table 2, the values presented in parentheses after the mean and standard deviation are not clear. It is recommended to add a note at the end of the table to clarify this aspect and provide a proper understanding of the data.

We appreciate the reviewer's comment. The values within the parentheses represent the median values. After careful consideration, we have decided to remove these values from table 2. This decision was made because the median values closely aligned with the mean values and did not significantly contribute to the results. This change also eliminates unnecessary complexity. Consequently, we have removed the median values from Tables 3, S1, S4, and S5 as well.

Another important point is the lack of consistency in table presentation. Some tables follow one format, while others have a different style. Improving this aspect to achieve a more consistent and professional presentation is suggested.

We have thoroughly reviewed the tables and have made the necessary adjustments to ensure consistency in the formatting of the tables.

The authors should also consider providing complete information about the validated instrument or

questionnaire, including the questions and response options. This would enable a better understanding of the tool used in the research.

Thank you for this suggestion, we added a supplement file with a translation of the FFQ used in our study. (File S1)

The information presented at the end of Table 4, specifically the median, is not clear. The authors should review if there is any error in the calculation of this descriptive statistic and correct it as needed. Furthermore, an excessive number of tables are observed in the manuscripts. It would be advisable to consider the possibility of consolidating information into one table or expressing some data in text form to simplify the presentation.

Following your suggestion, we merged table 4 and table S5. removed the median values, and improved the presentation of information in this table to enhance clarity.

Finally, it is suggested that one of the graphs be eliminated, and the information it contains be presented in text form, which could enhance overall clarity and presentation.

Following your suggestion, we removed figure 1 that presented the distribution of food items in the new and original FFQ.

References

1. Zheng M, Campbell KJ, Scanlan E, McNaughton SA. Development and evaluation of a food frequency questionnaire for use among young children. Moran JM, editor. PLoS ONE. 2020;15: e0230669. doi:10.1371/journal.pone.0230669

Beverley Yohanan
English Editing & Copywriting

November 5, 2023

To whom it may concern,

I would like to inform you that I have edited the article entitled “Development and validation of the MY-VEG-FFQ: A modular web-based food-frequency questionnaire for vegetarians and vegans” for my clients.

I have vast experience editing academic articles in a range of fields, many of which have been published in a variety of journals over the years.

For more information, please visit my website at www.business-letters.co.il or contact me at info@business-letters.co.il

Yours sincerely,

Beverely Yohanan

052-7702233 info@business-letters.co.il ● www.business-letters.co.il PO Box 459, Givat Ella 3657000
