# Supplementary material for: Development and validation of the MY-VEG-FFQ: A modular web-based food-frequency questionnaire for vegetarians and vegans
Source: PLoS One. 2024 Apr 16;19(4):e0299515. doi: 10.1371/journal.pone.0299515 (PMC11020715; doi:10.1371/journal.pone.0299515)
Supplement: Attachment — Submitted filename: Response to Reviewers.pdf [file pone.0299515.s014.pdf]

Ms. No. PONE-D-23-25091

Development and validation of the MY-VEG-FFQ: A modular web-based food-frequency questionnaire for vegetarians and vegans

To: **Diego A. Bonilla**
Academic Editor
PLOS ONE

Dear Dr. Bonilla,

We express our gratitude once again to you and the reviewers for your valuable contributions and the time dedicated to providing feedback on our work. Below, you will find the editor's comments along with our corresponding responses.

We hope that the manuscript in its current state meets the criteria for acceptance in the PLOS ONE journal.

Thank you for your reconsideration.

Sincerely,

Prof. Danit R Shahar, RD, PhD

The International center of health innovation & Nutrition

Department of Epidemiology, Biostatistics and Community Health Sciences

School of Public Health, Faculty of Health Sciences

Ben-Gurion University of the Negev, Israel

Point by point response:

1. The English language requires improvement throughout the manuscript. For example, the authors state the word via 2 ties in this sentence: "The validation analysis showed that nutrient-intake estimates were generally higher via the MY-VEG-FFQ than via the three-day food records". Thorough proofreading by a native English speaker is recommended to enhance grammar (authors might attach the translation certificate for transparency and quality).

The manuscript has undergone professional re-editing by the expert staff of American Manuscript Editors. Attached to this file, is their English editing certificate. We hope the re-edited version meets your expectations.

2. Although "body weight", "height" and "gender" are frequently used terms, it is technically correct to refer to "body mass", "stature" and "sex", respectively. Please address this accordingly through the manuscript.

We changed the terms body weight", "height" and "gender" to body mass", "stature" and "sex", respectively (lines 94, 232 and tables 1,3, S4, S5).

3. Enhance the quality of the manuscript by following the STROBE guidelines for structuring the text and sections. For example, the subsection "sample size" should be before the "Statistical analysis". Attach the checklist accordingly, for example, https://www.strobe-statement.org/checklists/ - Cf, www.equator-network.org

We implemented the required changes to adhere to the STROBE guidelines and shifted the subsection on sample size ahead of the statistical analysis (lines 163-170).

4. Do not use "mean±standard deviation". Use Mean(SD) instead. Cf, PMID 21206631

We converted the mean ± SD to mean (SD) throughout the manuscript, including in all the tables and supplements (lines 203, 204, 240, 241 and tables 1, 2, 3, S1, S4, S5)

5. Replace "Consequently, most analysis was computed using nonparametric tests or natural log transformations to increase normality" with "Consequently, most analyses were performed using nonparametric tests or natural log transformations to achieve normality."

We changed the sentence as requested (lines 177, 178)

6. The symbol of kilogram is kg not Kg. Revise units according to SI.

We changed the units to kg (table 1)

7. Be cautious with the interpretation and generalizability of the findings.

We added a remark regarding the interpretation and generalizability of the findings (line 339)

American Manuscript Editors

English Editing Certificate

This document certifies that the manuscript listed below was edited by the expert staff of American Manuscript Editors, all of whom are native English speakers. Moreover, the document was edited for proper English language, grammar, punctuation, and spelling.

Manuscript Title:

Development and validation of the MY-VEG-FFQ: A modular web-based food-frequency questionnaire for vegetarians and vegans

Authors:

Kerem Avital, Sigal Tepper, Sivan Ben-Avraham, Danit Rivka Shahar

Certificate Verification Key:

714-625-365-943-007

Project Number:

99819

This certificate may be verified by emailing info@americanmanuscripteditors.com. Documents receiving this certificate should be prepared for publication. However, please note that the author has the ability to accept or reject our suggestions for changes and can make changes after the editing process is complete, all of which can adversely affect the quality of the text after the editing process.

AMERICAN MANUSCRIPT EDITORS
